# 4-[(*E*)-4-Bromo­benzyl­ideneamino]-3-methyl-1*H*-1,2,4-triazole-5(4*H*)-thione

**DOI:** 10.1107/S1600536808021636

**Published:** 2008-07-16

**Authors:** Hoong-Kun Fun, Samuel Robinson Jebas, K. V. Sujith, P. S. Patil, B. Kalluraya, A. Muralidharan, S. M. Dharmaprakash

**Affiliations:** a X-ray Crystallography Unit, School of Physics, Universiti Sains Malaysia, 11800 USM, Penang, Malaysia; b Department of Studies in Chemistry, Mangalore University, Mangalagangotri, Mangalore 574 199, India; c Department of Studies in Physics, Mangalore University, Mangalagangotri, Mangalore 574 199, India; d Department of Chemistry, Nehru Arts and Science College, Kanhangad, Kerala 671 328, India

## Abstract

In the title mol­ecule, C_10_H_9_BrN_4_S, the dihedral angle between the triazole and benzene rings is 12.32 (19)°. An intra­molecular C—H⋯S hydrogen bond generates an *S*(6) ring motif. In the crystal packing, centrosymmetrically related mol­ecules are linked into a dimer by N—H⋯S hydrogen bonds, and the dimers are linked into a chain running along [1
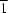
1] by Br⋯N short contacts [3.187 (3) Å]. The crystal packing is further strengthened by π–π inter­actions involving the triazole ring [centroid–centroid distance = 3.322 (2) Å].

## Related literature

For the pharmacological activity of triazole compounds, see: Bekircan *et al.* (2006 ▶); Brandt *et al.* (2007 ▶); Holla *et al.* (1996 ▶, 2002 ▶); Yale *et al.* (1966 ▶). For bond-length data, see: Allen *et al.* (1987 ▶). For graph-set analysis of hydrogen bonding, see: Bernstein *et al.* (1995 ▶).
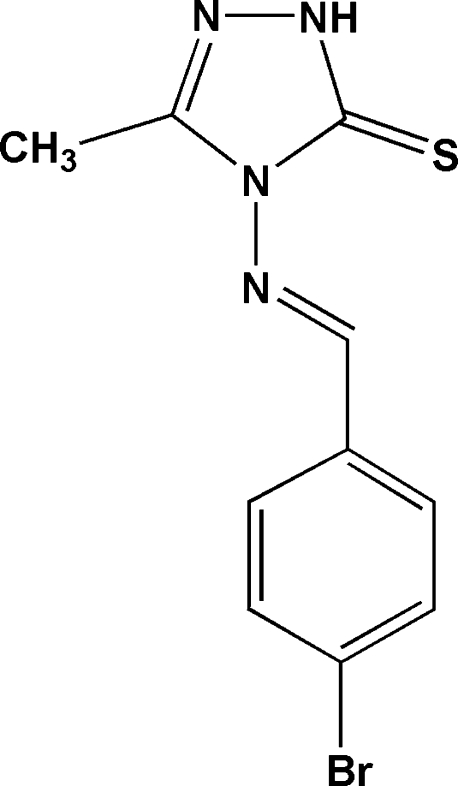


## Experimental

### 

#### Crystal data

C_10_H_9_BrN_4_S
*M* *_r_* = 297.18
Triclinic, 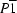

*a* = 6.9239 (5) Å
*b* = 7.6072 (5) Å
*c* = 11.5982 (8) Å
α = 82.453 (5)°
β = 88.339 (5)°
γ = 68.204 (4)°
*V* = 562.18 (7) Å^3^
*Z* = 2
Mo *K*α radiation
μ = 3.82 mm^−1^
*T* = 100.0 (1) K
0.32 × 0.31 × 0.12 mm

#### Data collection

Bruker SMART APEXII CCD area-detector diffractometer
Absorption correction: multi-scan (*SADABS*; Bruker, 2005 ▶) *T*_min_ = 0.265, *T*_max_ = 0.629
13535 measured reflections
3252 independent reflections
2538 reflections with *I* > 2σ(*I*)
*R*_int_ = 0.059

#### Refinement

*R*[*F* ^2^ > 2σ(*F* ^2^)] = 0.046
*wR*(*F* ^2^) = 0.121
*S* = 1.09
3252 reflections
146 parameters
H-atom parameters constrained
Δρ_max_ = 1.20 e Å^−3^
Δρ_min_ = −1.50 e Å^−3^

### 

Data collection: *APEX2* (Bruker, 2005 ▶); cell refinement: *APEX2*; data reduction: *SAINT* (Bruker, 2005 ▶); program(s) used to solve structure: *SHELXTL* (Sheldrick, 2008 ▶); program(s) used to refine structure: *SHELXTL*; molecular graphics: *SHELXTL*; software used to prepare material for publication: *SHELXTL* and *PLATON* (Spek, 2003 ▶).

## Supplementary Material

Crystal structure: contains datablocks global, I. DOI: 10.1107/S1600536808021636/ci2629sup1.cif

Structure factors: contains datablocks I. DOI: 10.1107/S1600536808021636/ci2629Isup2.hkl

Additional supplementary materials:  crystallographic information; 3D view; checkCIF report

## Figures and Tables

**Table 1:** Hydrogen-bond geometry (Å, °)

*D*—H⋯*A*	*D*—H	H⋯*A*	*D*⋯*A*	*D*—H⋯*A*
N3—H1*N*3⋯S1^i^	0.87	2.48	3.321 (4)	164
C7—H7*A*⋯S1	0.93	2.50	3.223 (4)	134

Symmetry code: (i) 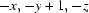.

**Fig. 1.**
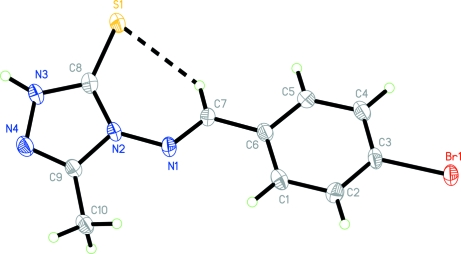
The molecular structure of the title compound, showing 50% probability displacement ellipsoids and the atom-numbering scheme. The dashed line indicates a hydrogen bond.

**Fig. 2.**
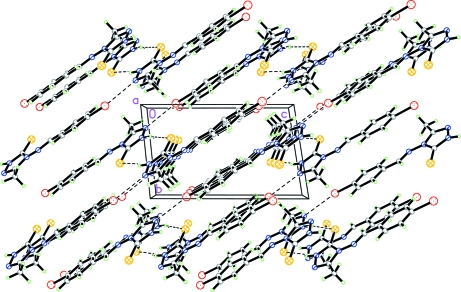
The crystal packing of the title compound, viewed along the a axis. Hydrogen bonds and Br···N short contacts are shown as dashed lines.

## supplementary crystallographic information

### Comment

Various 1,2,4-triazole derivatives are found to be associated with diverse pharmacological activity (Holla *et al.*, 1996,2002). Schiff bases of 1,2,4-triazoles find diverse applications and extensive biological activity. Schiff bases derived from 3-substituted-4-amino-5-mercapto-1,2,4 triazoles show antiinflammatory, analgesic, antimicrobial and antidepressant activities (Yale *et al.*, 1966; Bekircan *et al.*, 2006). The incorporation of the 1,2,4-triazole unit into Schiff-base macrocycles is of considerable current interest as complexes of 1,2,4-triazoles are being developed for potential use in applications such as magnetic materials and photochemically driven molecular devices (Brandt *et al.*, 2007). These applications prompted us to synthesize a novel Schiff base, derived from the reaction of 4-amino-5-methyl-2,4-dihydro-3*H*-1,2,4- triazole-3-thione with 4-bromo benzaldehyde.

In the title compound (Fig.1), the bond lengths and angles are found to have normal values (Allen *et al.*, 1987). The dihedral angle between the triazole ring (N2/C8/N3/N4/C9) and the benzene ring (C1-C6) is 12.32 (19)°, indicating that they are slightly twisted from each other. An intramolecular C—H···S hydrogen bond generates an S(6) ring motif (Bernstein *et al.*, 1995).

In the crystal packing, centrosymmetrically related molecules are linked into a dimer by N—H···S hydrogen bonds (Table 1). The dimers are linked into a chain running along the [1 1 1] by Br1···N4(1+x, -1+y, 1+z) short contacts [3.187 (3) Å]. The crystal packing is further strengthened by π-π interactions between the N2/C8/N3/N4/C9 (centroid *Cg*1) rings of the molecules at (x, y, z) and (1-x, 1-y, z) [centroid-centroid distance = 3.322 (2) Å].

### Experimental

A mixture of 4-amino-5-methyl-2,4-dihydro-3*H*-1,2,4-triazole-3-thione (0.01 mol), 4-bromobenzaldehyde (0.01 mol) in ethanol (30 ml) and 2 drops of concentrated H_2_SO_4_ was refluxed for 3 h. The solid product obtained was collected by filtration, washed with ethanol and dried. Single crystals suitable for X-ray analysis were obtained from ethanol by slow evaporation.

### Refinement

H atoms were positioned geometrically [C-H = 0.93-0.96 %A and N-H = 0.87 Å] and refined using a riding model, with *U*_iso_(H) = 1.2*U*_eq_(C,N) and 1.5_eq_(C_methyl_). A rotating group model was used for the methyl groups.

### Crystal data

**Table:**

C_10_H_9_BrN_4_S	*Z* = 2
*M**_r_* = 297.18	*F*_000_ = 296
Triclinic, *P*1	*D*_x_ = 1.756 Mg m^−^^3^
Hall symbol: -P 1	Mo *K*α radiation λ = 0.71073 Å
*a* = 6.9239 (5) Å	Cell parameters from 5175 reflections
*b* = 7.6072 (5) Å	θ = 2.9–33.2º
*c* = 11.5982 (8) Å	µ = 3.82 mm^−^^1^
α = 82.453 (5)º	*T* = 100.0 (1) K
β = 88.339 (5)º	Plate, colourless
γ = 68.204 (4)º	0.32 × 0.31 × 0.12 mm
*V* = 562.18 (7) Å^3^	

### Data collection

**Table:**

Bruker SMART APEXII CCD area-detector diffractometer	3252 independent reflections
Radiation source: fine-focus sealed tube	2538 reflections with *I* > 2σ(*I*)
Monochromator: graphite	*R*_int_ = 0.059
*T* = 100.0(1) K	θ_max_ = 30.0º
φ and ω scans	θ_min_ = 1.8º
Absorption correction: multi-scan(SADABS; Bruker, 2005)	*h* = −9→9
*T*_min_ = 0.265, *T*_max_ = 0.629	*k* = −10→10
13535 measured reflections	*l* = −16→16

### Refinement

**Table:**

Refinement on *F*^2^	Secondary atom site location: difference Fourier map
Least-squares matrix: full	Hydrogen site location: inferred from neighbouring sites
*R*[*F*^2^ > 2σ(*F*^2^)] = 0.046	H-atom parameters constrained
*wR*(*F*^2^) = 0.121	*w* = 1/[σ^2^(*F*_o_^2^) + (0.0635*P*)^2^ + 0.1826*P*] where *P* = (*F*_o_^2^ + 2*F*_c_^2^)/3
*S* = 1.09	(Δ/σ)_max_ = 0.001
3252 reflections	Δρ_max_ = 1.20 e Å^−^^3^
146 parameters	Δρ_min_ = −1.50 e Å^−^^3^
Primary atom site location: structure-invariant direct methods	Extinction correction: none

### Special details

**Table:**

Experimental. The data was collected with the Oxford Cyrosystem Cobra low-temperature attachment.
Geometry. All e.s.d.'s (except the e.s.d. in the dihedral angle between two l.s. planes) are estimated using the full covariance matrix. The cell e.s.d.'s are taken into account individually in the estimation of e.s.d.'s in distances, angles and torsion angles; correlations between e.s.d.'s in cell parameters are only used when they are defined by crystal symmetry. An approximate (isotropic) treatment of cell e.s.d.'s is used for estimating e.s.d.'s involving l.s. planes.
Refinement. Refinement of *F*^2^ against ALL reflections. The weighted *R*-factor *wR* and goodness of fit *S* are based on *F*^2^, conventional *R*-factors *R* are based on *F*, with *F* set to zero for negative *F*^2^. The threshold expression of *F*^2^ > σ(*F*^2^) is used only for calculating *R*-factors(gt) *etc*. and is not relevant to the choice of reflections for refinement. *R*-factors based on *F*^2^ are statistically about twice as large as those based on *F*, and *R*- factors based on ALL data will be even larger.

### Fractional atomic coordinates and isotropic or equivalent isotropic displacement parameters (Å^2^)

**Table:**

	*x*	*y*	*z*	*U*_iso_*/*U*_eq_	
Br1	1.07281 (6)	0.01690 (5)	0.76969 (3)	0.02379 (13)	
S1	0.13495 (15)	0.36883 (13)	0.17836 (8)	0.0247 (2)	
N1	0.5353 (5)	0.4917 (4)	0.2620 (2)	0.0212 (6)	
N2	0.4008 (5)	0.5702 (4)	0.1661 (2)	0.0201 (6)	
N3	0.1919 (5)	0.6408 (4)	0.0225 (3)	0.0243 (6)	
N4	0.3110 (5)	0.7506 (4)	−0.0027 (3)	0.0236 (6)	
C1	0.8184 (6)	0.3339 (5)	0.4542 (3)	0.0237 (7)	
H1A	0.8534	0.4121	0.3957	0.028*	
C2	0.9486 (6)	0.2514 (5)	0.5516 (3)	0.0246 (7)	
H2A	1.0698	0.2751	0.5590	0.029*	
C3	0.8955 (6)	0.1335 (5)	0.6374 (3)	0.0214 (7)	
C4	0.7153 (6)	0.0956 (5)	0.6288 (3)	0.0230 (7)	
H4A	0.6830	0.0140	0.6862	0.028*	
C5	0.5843 (6)	0.1829 (5)	0.5321 (3)	0.0224 (7)	
H5A	0.4606	0.1626	0.5265	0.027*	
C6	0.6348 (5)	0.2997 (5)	0.4439 (3)	0.0199 (7)	
C7	0.4924 (5)	0.3838 (5)	0.3443 (3)	0.0199 (7)	
H7A	0.3710	0.3592	0.3407	0.024*	
C8	0.2417 (6)	0.5275 (5)	0.1234 (3)	0.0217 (7)	
C9	0.4376 (6)	0.7049 (5)	0.0860 (3)	0.0211 (7)	
C10	0.5990 (6)	0.7828 (5)	0.1022 (3)	0.0249 (7)	
H10A	0.6335	0.8346	0.0280	0.037*	
H10B	0.7209	0.6828	0.1377	0.037*	
H10C	0.5478	0.8818	0.1514	0.037*	
H1N3	0.1157	0.6542	−0.0384	0.030*	

### Atomic displacement parameters (Å^2^)

**Table:**

	*U*^11^	*U*^22^	*U*^33^	*U*^12^	*U*^13^	*U*^23^
Br1	0.0287 (2)	0.02116 (18)	0.01825 (18)	−0.00691 (14)	−0.00647 (13)	0.00315 (12)
S1	0.0301 (5)	0.0264 (4)	0.0191 (4)	−0.0150 (4)	−0.0050 (3)	0.0069 (3)
N1	0.0242 (15)	0.0186 (13)	0.0177 (14)	−0.0058 (12)	−0.0047 (11)	0.0029 (11)
N2	0.0251 (14)	0.0185 (13)	0.0154 (13)	−0.0085 (12)	−0.0020 (11)	0.0041 (10)
N3	0.0318 (16)	0.0226 (14)	0.0179 (14)	−0.0120 (13)	−0.0029 (12)	0.0058 (11)
N4	0.0259 (15)	0.0242 (15)	0.0203 (14)	−0.0109 (13)	0.0003 (12)	0.0040 (11)
C1	0.0278 (18)	0.0222 (16)	0.0194 (16)	−0.0094 (14)	0.0024 (14)	0.0028 (13)
C2	0.0227 (17)	0.0252 (17)	0.0243 (18)	−0.0076 (14)	−0.0052 (14)	−0.0009 (14)
C3	0.0253 (17)	0.0167 (15)	0.0164 (15)	−0.0024 (13)	−0.0035 (13)	0.0022 (12)
C4	0.0307 (19)	0.0178 (15)	0.0188 (16)	−0.0083 (14)	0.0018 (14)	0.0007 (12)
C5	0.0255 (17)	0.0198 (16)	0.0225 (17)	−0.0100 (14)	−0.0034 (14)	0.0002 (13)
C6	0.0237 (16)	0.0176 (15)	0.0173 (15)	−0.0074 (13)	−0.0001 (13)	0.0003 (12)
C7	0.0239 (16)	0.0191 (15)	0.0158 (15)	−0.0079 (13)	−0.0044 (13)	0.0009 (12)
C8	0.0229 (16)	0.0210 (16)	0.0182 (16)	−0.0057 (14)	−0.0004 (13)	0.0006 (12)
C9	0.0252 (17)	0.0181 (15)	0.0195 (16)	−0.0088 (14)	−0.0006 (13)	0.0013 (12)
C10	0.0285 (18)	0.0230 (17)	0.0228 (17)	−0.0116 (15)	−0.0006 (14)	0.0047 (13)

### Geometric parameters (Å, °)

**Table:**

Br1—C3	1.895 (3)	C2—C3	1.386 (5)
S1—C8	1.686 (4)	C2—H2A	0.93
N1—C7	1.278 (4)	C3—C4	1.390 (5)
N1—N2	1.390 (4)	C4—C5	1.392 (5)
N2—C8	1.380 (5)	C4—H4A	0.93
N2—C9	1.381 (4)	C5—C6	1.390 (5)
N3—C8	1.331 (4)	C5—H5A	0.93
N3—N4	1.377 (4)	C6—C7	1.455 (4)
N3—H1N3	0.87	C7—H7A	0.93
N4—C9	1.296 (5)	C9—C10	1.473 (5)
C1—C2	1.391 (5)	C10—H10A	0.96
C1—C6	1.400 (5)	C10—H10B	0.96
C1—H1A	0.93	C10—H10C	0.96
			
C7—N1—N2	119.6 (3)	C6—C5—H5A	119.4
C8—N2—C9	108.5 (3)	C4—C5—H5A	119.4
C8—N2—N1	133.0 (3)	C5—C6—C1	119.2 (3)
C9—N2—N1	118.1 (3)	C5—C6—C7	118.3 (3)
C8—N3—N4	114.1 (3)	C1—C6—C7	122.5 (3)
C8—N3—H1N3	137.0	N1—C7—C6	119.6 (3)
N4—N3—H1N3	108.1	N1—C7—H7A	120.2
C9—N4—N3	104.3 (3)	C6—C7—H7A	120.2
C2—C1—C6	120.3 (3)	N3—C8—N2	102.7 (3)
C2—C1—H1A	119.8	N3—C8—S1	126.6 (3)
C6—C1—H1A	119.8	N2—C8—S1	130.6 (3)
C3—C2—C1	119.2 (4)	N4—C9—N2	110.4 (3)
C3—C2—H2A	120.4	N4—C9—C10	126.1 (3)
C1—C2—H2A	120.4	N2—C9—C10	123.5 (3)
C2—C3—C4	121.7 (3)	C9—C10—H10A	109.5
C2—C3—Br1	119.8 (3)	C9—C10—H10B	109.5
C4—C3—Br1	118.5 (3)	H10A—C10—H10B	109.5
C3—C4—C5	118.4 (3)	C9—C10—H10C	109.5
C3—C4—H4A	120.8	H10A—C10—H10C	109.5
C5—C4—H4A	120.8	H10B—C10—H10C	109.5
C6—C5—C4	121.2 (3)		
			
C7—N1—N2—C8	−16.6 (6)	C5—C6—C7—N1	−179.9 (3)
C7—N1—N2—C9	171.9 (3)	C1—C6—C7—N1	0.7 (5)
C8—N3—N4—C9	−0.5 (4)	N4—N3—C8—N2	0.9 (4)
C6—C1—C2—C3	0.7 (5)	N4—N3—C8—S1	−177.3 (3)
C1—C2—C3—C4	−0.1 (5)	C9—N2—C8—N3	−1.0 (4)
C1—C2—C3—Br1	179.1 (3)	N1—N2—C8—N3	−173.0 (3)
C2—C3—C4—C5	−1.4 (5)	C9—N2—C8—S1	177.2 (3)
Br1—C3—C4—C5	179.4 (3)	N1—N2—C8—S1	5.1 (6)
C3—C4—C5—C6	2.3 (5)	N3—N4—C9—N2	−0.2 (4)
C4—C5—C6—C1	−1.7 (5)	N3—N4—C9—C10	180.0 (3)
C4—C5—C6—C7	179.0 (3)	C8—N2—C9—N4	0.8 (4)
C2—C1—C6—C5	0.1 (5)	N1—N2—C9—N4	174.2 (3)
C2—C1—C6—C7	179.5 (3)	C8—N2—C9—C10	−179.4 (3)
N2—N1—C7—C6	179.2 (3)	N1—N2—C9—C10	−6.0 (5)

### Hydrogen-bond geometry (Å, °)

**Table:**

*D*—H···*A*	*D*—H	H···*A*	*D*···*A*	*D*—H···*A*
N3—H1N3···S1^i^	0.87	2.48	3.321 (4)	164
C7—H7A···S1	0.93	2.50	3.223 (4)	134

Symmetry codes: (i) −*x*, −*y*+1, −*z*.
